# Pre-Season Changes in Physical Performance and Early Competitive Squad Inclusion in Under-15 Male Soccer Players

**DOI:** 10.3390/jfmk11030300

**Published:** 2026-07-29

**Authors:** Artur Avelino Birk Preissler, Filipe Manuel Clemente, Júlio Brugnara Mello, Ana Filipa Silva, Rui Miguel Silva, Pedro Schons

**Affiliations:** 1 Educação Física, Faculdade SOGIPA, Porto Alegre 90240-485, Brazil; pedroschons@hotmail.com; 2 School of Physical Education, Physiotherapy and Dance, Federal University of Rio Grande do Sul, Porto Alegre 90690-200, Brazil; 3 Applied Research Institute (i2A), Polytechnic University of Coimbra, 3045-093 Coimbra, Portugal; filipe.clemente5@gmail.com; 4 The Sport Physical Activity and Health Research & Innovation Center, 3045-093 Coimbra, Portugal; 5 Department of Biomechanics and Sport Engineering, Gdansk University of Physical Education and Sport, 80-336 Gdańsk, Poland; 6 Grupo de Investigación en Adaptaciones Musculoesqueléticas al Entrenamiento, Physical Education School, Pontificia Universidad Católica de Valparaíso, Valparaíso 2340025, Chile; julio.mello@pucv.cl; 7 Faculty of Sport Sciences and Physical Education, University of Coimbra, 3040-256 Coimbra, Portugal; anafilsilva@gmail.com; 8 Escola Superior de Desporto e Lazer, Instituto Politécnico de Viana do Castelo, 4900-347 Viana do Castelo, Portugal; rui.s@ipvc.pt; 9 The Sport Physical Activity and Health Research & Innovation Center, 4900-347 Viana do Castelo, Portugal

**Keywords:** youth soccer, physical performance, pre-season, squad inclusion, change in direction, biological maturation

## Abstract

**Background**: Pre-season physical monitoring may help coaches understand short-term changes in youth soccer players and examine whether physical indicators are associated with early squad inclusion. This study aimed to analyze changes in physical performance during the pre-season period and to examine whether pre- and post-assessment physical performance differed between squad-included and non-included under-15 male soccer players in the first five official matches of the main competition. **Methods**: Thirty-seven male outfield players were assessed using the Squat Jump (SJ), Countermovement Jump (CMJ), 20 m linear sprint (Sprint-20 m), 20 m change-in-direction test (COD-20 m), and Yo-Yo Intermittent Recovery Test Level 1 (Yo-Yo IRL1). Squad-included players were those listed in at least one of the first five official match reports, whereas non-included players were not listed in these reports but remained part of the broader under-15 program. **Results**: Significant increases were observed in SJ (+1.44 cm, *p* = 0.007), CMJ (+1.36 cm, *p* = 0.006), and COD-20 m (+0.38 km·h^−1^, *p* = 0.002), whereas Sprint-20 m and Yo-Yo IRL1 did not change significantly. Fourteen players were squad-included and 23 were non-included. Squad-included players presented higher pre-assessment values for SJ, CMJ, COD-20 m, and the exploratory composite physical score, and higher post-assessment values for SJ, CMJ, Sprint-20 m, COD-20 m, and the exploratory composite physical score. The largest between-group difference was observed for post-assessment COD-20 m (14.19 ± 0.49 vs. 13.40 ± 0.74 km·h^−1^, *p* < 0.001, d = 1.25). **Conclusions**: Early squad inclusion was associated with higher absolute physical performance, particularly post-assessment COD-20 m, whereas the magnitude of pre-to-post changes was not clearly associated with squad inclusion.

## 1. Introduction

In youth soccer, talent development and early selection decisions operate within a multidimensional performance system in which technical, tactical, physical, psychological, biological, and contextual factors interact across adolescence [1,2]. Although physical attributes are only one component of this system, objective field testing is commonly used to complement coach observation because jumping, sprinting, change-in-direction, and intermittent-running capacities are measurable, trainable, and relevant to player profiling [3,4]. The value of such monitoring depends on interpreting test outcomes as information about current physical readiness rather than as stand-alone evidence of talent or long-term potential [2,5].

Within this framework, the pre-season period is a practical window for baseline profiling because short preparation blocks in young soccer players have used repeated testing of sprinting, jumping, and change-in-direction performance to evaluate physical adaptation [6]. Intermittent running capacity is one of the relevant tests because the Yo-Yo Intermittent Recovery Test Level 1 evaluates the ability to repeatedly perform intense intermittent exercise and is widely used in sports with intermittent activity profiles [7,8]. Moreover, strength and power are also relevant physical capacities that support the high-speed and powerful actions [9]. A testing battery that combines Squat Jump, Countermovement Jump, linear sprinting, change-in-direction, and Yo-Yo Intermittent Recovery Test Level 1 outcomes therefore captures complementary domains of youth soccer physical readiness [4,10,11].

Prior evidence suggests that physical and physiological measures can contribute to youth soccer prognosis and selection, but their effects are heterogeneous and usually modest rather than deterministic [3]. Field-based fitness tests in youth soccer show acceptable reliability and can discriminate between players with different pathway status, although reliability and interpretation may vary by test and age group [4]. Earlier selection studies reported that selected youth players were often taller, leaner, faster, and more aerobically fit than non-selected peers [12]. Biological maturation is especially relevant during adolescence because maturity status is associated with anthropometric and physical-fitness variables in young athletes [13]. In soccer academy, maturity has been associated with sprint performance and with selection bias independently of chronological age or relative age [14,15].

Recent youth soccer studies have connected biological and physical indicators with competitive participation, including under-15 research combining maturity, fitness, and hormonal measures and a pre-season study relating physical assessments to subsequent playing time [16,17]. However, playing time across broader follow-up periods does not answer whether players included in the first official squad lists after pre-season differ in absolute physical performance, short-term physical change, or estimated biological maturation [5]. This distinction is important because change scores can be influenced by baseline status, measurement variability, growth and maturation, and training exposure [4,18]. Official squad inclusion is also an applied competitive decision embedded in technical, tactical, positional, medical, psychological, and contextual information [1,5]. Clarifying the relationship between pre-season physical indicators and early squad inclusion can help practitioners use testing data to inform readiness discussions without reducing inclusion decisions to physical performance alone [2,5].

Therefore, associating pre-season physical assessments with early official match-report squad listings offers a way to examine the applied relevance of physical monitoring at the transition from preparation to competition [4,5]. The present study aimed to analyze changes in physical performance during the pre-season period and to examine whether pre- and post-assessment physical performance differed between squad-included and non-included under-15 male soccer players in the first five official matches of the main competition. As complementary aims, the study examined associations between physical performance and the number of squad inclusions and explored whether estimated maturity offset accompanied early competitive involvement. We hypothesized that squad-included players would present superior absolute physical performance, particularly at post-assessment, whereas pre-to-post changes and estimated maturity offset would show weaker associations with early squad inclusion.

## 2. Materials and Methods

### 2.1. Participants

Participants were male outfield soccer players from an under-15 youth soccer program. This study was designed as an observational, longitudinal, descriptive, and quantitative investigation conducted during the pre-season period. Participants were recruited using a convenience sampling approach, based on their availability to complete the physical testing battery at both assessment moments and on the availability of official match reports from the first five competitive matches of the main state competition analyzed in the present study. The final sample consisted of 37 players, including forwards (*n* = 11), midfielders (*n* = 11), center-backs (*n* = 9), and full-backs (*n* = 6). At baseline, players were 13.76 ± 0.60 years old, had 7.08 ± 1.95 years of soccer practice, stature of 170.16 ± 8.20 cm, sitting height of 87.57 ± 4.75 cm, body mass of 59.40 ± 9.09 kg, estimated maturity offset of 0.33 ± 0.76 years from peak height velocity. Goalkeepers were excluded due to the specific physical and technical demands of their position. Players without complete paired data in the pre- and post-assessment moments were also excluded from the analysis. Competitive involvement was determined from the official match reports of the first five official matches of the main competition. Players were classified as squad-included when they were listed in the official squad in at least one of the five matches, regardless of whether they started the match or were listed as substitutes. Players who were not listed in any of the five official match reports analyzed were classified as non-included. Based on this criterion, 14 players were classified as squad-included and 23 as non-included. Among squad-included players, there were five forwards, six midfielders, no center-backs, and three full-backs; among non-included players, there were six forwards, five midfielders, nine center-backs, and three full-backs. Importantly, non-included players remained part of the broader under-15 program and continued to participate in the team’s training process and competitive pathway, including opportunities in secondary fixtures or competitions within the club’s competitive structure. Therefore, the term non-included refers specifically to the absence of these players from the official squad lists of the five main competition matches analyzed, and should not be interpreted as dismissal from the team or exclusion from the training environment. The study was conducted in accordance with the principles of the Declaration of Helsinki. Written informed consent was obtained from the players’ legal guardians, and players provided assent before participation. Ethical approval was granted by the Research Ethics Committee of the Pontifical Catholic University of Rio Grande do Sul (CEP-PUCRS) (Approval number: 7.047.156; date of approval: 2 September 2024).

### 2.2. Design and Setting

**Figure 1:**
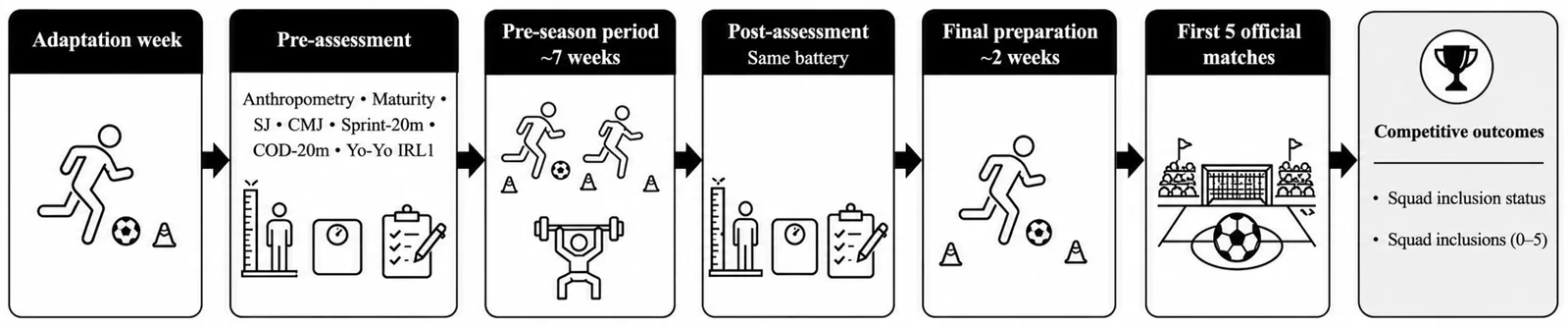
Study design and methodological flow of the pre-season period and competitive follow-up. The figure summarizes the sequence from the adaptation week to pre- and post-assessments, final preparation, the first five official matches, and the competitive outcomes analyzed. SJ = Squat Jump; CMJ = Countermovement Jump; COD-20 m = 20-m change-of-direction test; Yo-Yo IRL1 = Yo-Yo Intermittent Recovery Test Level 1.

Data were collected during the pre-season period in an applied youth soccer training setting. The team first completed an initial week of adaptation to the training routine. After this adaptation period, players performed the pre-assessment. The monitored pre-season period then lasted approximately seven weeks, during which players followed the team’s regular training routine, including technical, tactical, and physical components. The post-assessment was performed at the end of this monitored pre-season period. After the post-assessment, the team continued its final preparation phase, and the official competition began approximately two weeks later. The assessments were part of the regular physical performance monitoring routine and were conducted within the athletes’ training environment, aiming to minimize interference with the team’s schedule. The same testing procedures were adopted at both assessment moments. Before the physical tests, players performed a standardized warm-up composed of dynamic exercises commonly used in their training routine and received verbal instructions from the evaluators. The specific content, intensity, volume, and internal or external training loads of each session were not controlled or quantified in the present study. Competitive involvement was assessed after the beginning of the official competition using the official match reports from the first five matches of the main state competition. For each match, the official squad list was checked to identify whether each player was included or not. The primary competitive outcome was squad inclusion status, defined as squad-included or non-included. In addition, the number of squad inclusions across the five matches was calculated for each player, ranging from 0 to 5, and used as a complementary indicator of competitive involvement. The methodological flow of the study is summarized in Figure 1.

### 2.3. Procedures

The testing battery included: (i) anthropometric assessment; (ii) estimated maturity offset from peak height velocity; (iii) Squat Jump (SJ); (iv) Countermovement Jump (CMJ); (v) 20 m linear sprint (Sprint-20 m); (vi) 20 m change-in-direction test (COD-20 m); and (vii) Yo-Yo Intermittent Recovery Test Level 1 (Yo-Yo IRL1). The 20 m protocols were selected because they were established components of the team’s testing routine and enabled complementary assessment of linear sprint and change-in-direction performance over the same total distance. The same order of assessments was maintained at both moments to reduce variability related to the testing sequence. Anthropometric and maturational measures were obtained before the physical performance tests. Lower-limb power was assessed using SJ and CMJ. Linear sprint performance was assessed using Sprint-20 m, change-in-direction performance was assessed using COD-20 m, and intermittent running performance was assessed using Yo-Yo IRL1. For Sprint-20 m and COD-20 m, the fastest recorded time was converted to average velocity in km·h^−1^ using the equation: velocity = distance/time × 3.6. Consequently, higher values represented better performance in all physical tests analyzed. Because of the short interval between assessments, sample characterization and maturational interpretation were based only on pre-assessment values. Estimated maturity offset was used as a complementary indicator of biological maturation. The specific procedures for each test are described in the following subsections.

#### 2.3.1. Anthropometric Assessment

Anthropometric assessment included body mass, stature, and sitting height. Body mass was measured with a digital scale with 0.1 kg resolution (G-TECH, Accumed Produtos Médico Hospitalares Ltd.a., Duque de Caxias, Brazil), with players barefoot and wearing light clothing. Stature was measured using a non-extensible measuring tape fixed vertically to a flat wall, with 1 mm resolution. During stature measurement, players remained barefoot in an upright position, with heels together and head positioned in the Frankfurt plane. Sitting height was obtained using the same measuring tape, with players seated on a standardized bench, trunk upright, and head aligned in the Frankfurt plane. When required, bench height was subtracted to determine the distance from the sitting surface to the vertex of the head. Chronological age was calculated from the players’ date of birth and the date of the baseline assessment. Soccer practice experience was self-reported and recorded in years. Biological maturation was estimated using maturity offset from peak height velocity, according to the anthropometric prediction equation proposed by Mirwald et al. [19,20]. The equation includes chronological age, body mass, stature, sitting height, and leg length, with leg length calculated as stature minus sitting height. Maturity offset was expressed in years from peak height velocity, with negative values indicating that the player was before the estimated peak height velocity and positive values indicating that the player was after this maturational landmark.

#### 2.3.2. Vertical Jump Test

Lower-limb power was assessed using the Squat Jump (SJ) and Countermovement Jump (CMJ). Both tests were performed on a contact platform (Jump System, Cefise, Nova Odessa, Brazil) connected to a portable computer for data recording. Players performed all jumps with their hands placed on their hips to reduce the influence of arm swing on jump performance. For the SJ, players started from a static semi-squat position, with approximately 90° of knee flexion, and were instructed to jump as high as possible without performing a countermovement. For the CMJ, players started from an upright standing position and, after a verbal command, performed a rapid downward movement to approximately 90° of knee flexion, immediately followed by maximal lower-limb extension [21,22]. Two valid attempts were performed for each jump, with 30 s of passive recovery between attempts. The highest value obtained in each test was retained for analysis. Jump height was expressed in centimeters and calculated from flight time using the equation h = g × t^2^/8, where h represents jump height, g represents gravitational acceleration, and t represents flight time.

#### 2.3.3. Linear Sprint Tests

Linear sprint performance was assessed using the 20 m linear sprint test (Sprint-20 m). The test was performed on the field using an electronic timing system with photocells (CEFISE, São Paulo, Brazil), with 1 ms precision. Photocells were positioned at a height of 100 cm at the start and finish lines. Players began each trial from a standardized starting position, with the front foot placed 0.30 m behind the first timing gate and the trunk slightly inclined forward. After an auditory signal, players sprinted maximally over the 20 m distance. A cone was positioned 5 m beyond the final timing gate to encourage players to maintain maximal effort through the finish line and avoid premature deceleration [23,24]. Each player completed two trials, separated by 3 min of passive recovery. The fastest time was retained for analysis. Sprint performance was subsequently converted to average velocity in km·h^−1^ using the recorded time over the 20 m distance.

#### 2.3.4. Change-in-Direction Test

Change-in-direction performance was assessed using the 20 m change-in-direction test (COD-20 m). The test consisted of a 20 m zig-zag course composed of four 5 m sections, with cones positioned at 100° angles. Players were required to run around the outside of each cone and complete the course as quickly as possible. The test was conducted on the field using an electronic timing system with photocells (CEFISE, São Paulo, Brazil), positioned at a height of 100 cm at the start and finish lines. Players started from a standardized position, with the front foot placed 0.30 m behind the first timing gate. As in the linear sprint test, a cone was positioned 5 m beyond the finish line to reduce the likelihood of premature deceleration before crossing the final timing gate [23,24]. Each player performed two trials, with 3 min of passive recovery between attempts. The fastest time was retained for analysis. COD-20 m performance was then expressed as average velocity in km·h^−1^, calculated from the recorded time over the 20 m course.

#### 2.3.5. Yo-Yo Intermittent Recovery Test Level 1

Intermittent running performance was assessed using the Yo-Yo Intermittent Recovery Test Level 1 (Yo-Yo IRL1). The test consisted of repeated 2 × 20 m shuttle runs performed at progressively increasing speeds controlled by audio signals. Each running bout was followed by 10 s of active recovery over a 2 × 5 m area. Cones were used to mark the 20 m running lanes and the active recovery zone. Players were instructed to follow the rhythm dictated by the audio signals for as long as possible. The test was terminated when the player voluntarily stopped due to exhaustion or failed twice to reach the required line in synchrony with the audio signal. The total distance completed was recorded in meters and used as the outcome for intermittent running performance [7].

### 2.4. Statistical Analysis

Descriptive statistics were presented as mean ± standard deviation. Sample characterization was performed using only pre-assessment data. Individual change scores (Δ) were calculated as post-assessment minus pre-assessment values. Competitive involvement was determined from the official match reports of the first five matches. Players were classified as squad-included when they were listed in the official squad in at least one match and as non-included when they were not listed in any match. The number of squad inclusions across the five matches was also calculated and used as a complementary indicator of competitive involvement. The normality of the data was assessed using the Shapiro–Wilk test. For the overall pre-to-post comparisons, the normality of the change scores was considered. SJ and COD-20 m were analyzed using the Wilcoxon signed-rank test, whereas CMJ, Sprint-20 m, and Yo-Yo IRL1 were analyzed using paired *t*-tests. For between-group comparisons, squad-included and non-included players were compared using Welch’s *t*-test when variables satisfied the normality assumption, due to the unequal group sizes. For between-group comparisons of change scores, the Mann–Whitney U test was used when the normality assumption was not satisfied in at least one group. Cohen’s d was calculated for paired and Welch’s *t*-tests, whereas rank-biserial correlation was calculated for Wilcoxon signed-rank and Mann–Whitney U tests. A composite physical score was calculated as an exploratory standardized summary of the overall physical profile. SJ, CMJ, Sprint-20 m, COD-20 m, and Yo-Yo IRL1 were converted into z-scores using the pre-assessment mean and standard deviation as reference values. The composite physical score was calculated as the unweighted mean of the five z-scores at each assessment, and the composite change score was calculated as the post-assessment composite score minus the pre-assessment composite score. Equal weighting was adopted because there was no a priori empirical or theoretical basis for assigning different weights to the individual physical tests; accordingly, the score was interpreted only as an exploratory, non-validated summary measure. Associations between physical performance and competitive involvement were analyzed using Spearman’s rank correlation coefficient, because the number of squad inclusions was a discrete variable restricted to the first five official matches. Correlations were performed between the number of squad inclusions and the pre- and post-assessment values of each physical test and the composite physical score. As complementary analyses, Δ values were compared between squad-included and non-included players, and pre-assessment maturity offset from peak height velocity was compared between groups and correlated with the number of squad inclusions. The magnitudes of Spearman’s ρ were interpreted using absolute values as trivial (<0.10), small (0.10–0.29), moderate (0.30–0.49), large (0.50–0.69), very large (0.70–0.89), and nearly perfect (≥0.90) [25]. The significance level was set at α < 0.05. No adjustment for multiple comparisons was applied; therefore, the comparisons and correlations were interpreted as exploratory, with individual *p*-values considered alongside the magnitude and overall pattern of the findings. All analyses were performed using Jamovi software (version 2.6.26).

## 3. Results

Table 1 presents the changes in physical performance between pre- and post-assessments in the overall sample. Significant increases were observed in SJ, CMJ, and COD-20 m. No significant changes were observed in Sprint-20 m or Yo-Yo IRL1.

**Figure 2:**
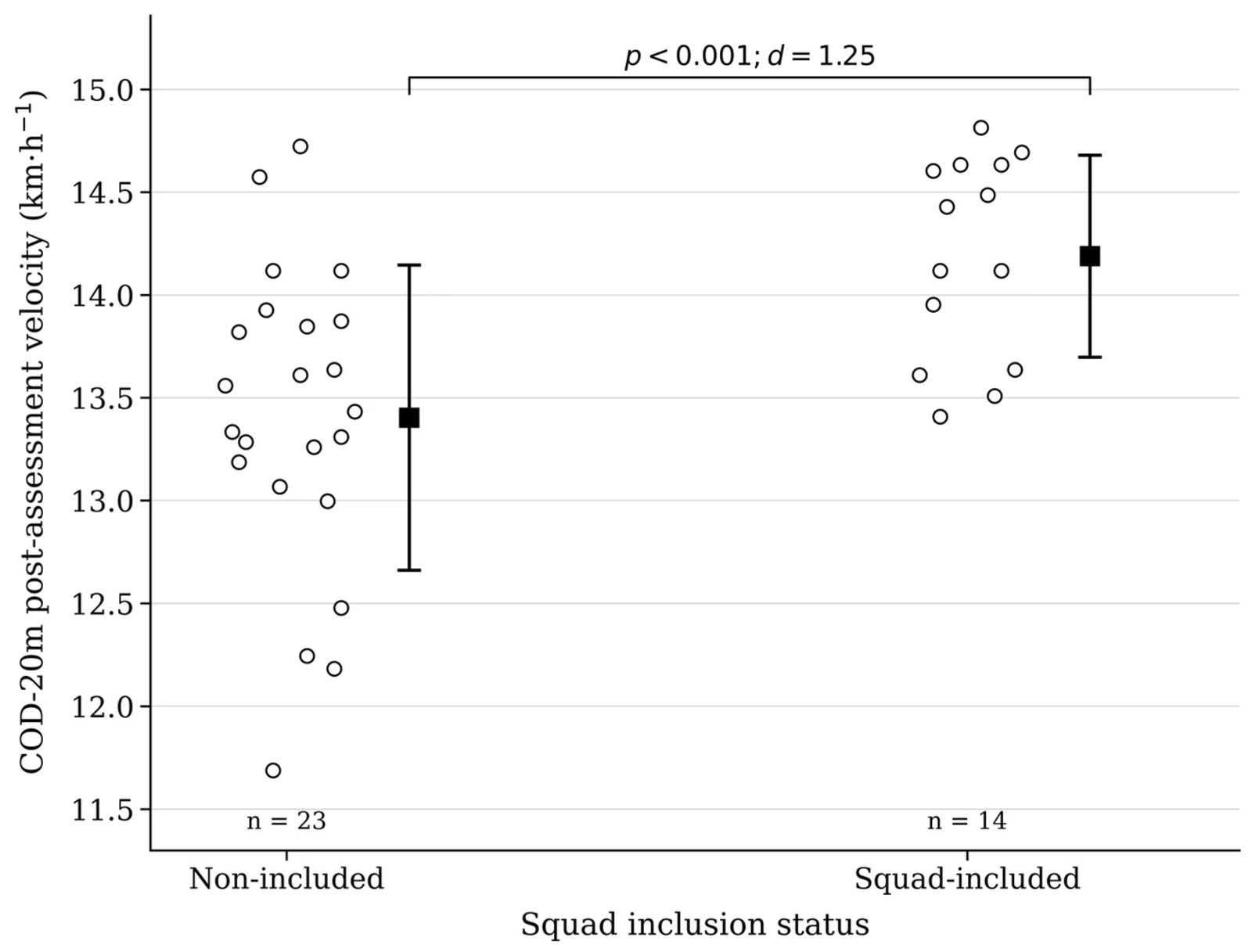
Post-assessment COD-20 m performance according to squad inclusion status. Open circles represent individual players, and black squares represent mean ± SD. COD-20 m was expressed as mean velocity (km·h^−1^); therefore, higher values indicate better change-in-direction performance. Squad-included players showed higher post-assessment COD-20 m values than non-included players (*p* < 0.001; d = 1.25).

Table 2 presents the comparison of pre- and post-assessment physical performance between squad-included and non-included players in the first five official matches. At pre-assessment, squad-included players presented higher values for SJ, CMJ, COD-20 m, and the composite physical score. At post-assessment, squad-included players presented higher values for SJ, CMJ, Sprint-20 m, COD-20 m, and the composite physical score. Yo-Yo IRL1 did not differ between squad-included and non-included players at either assessment. Figure 2 illustrates the individual distribution of post-assessment COD-20 m performance, which showed the largest between-group difference among the individual physical tests.

Table 3 presents, as a complementary analysis, the associations between absolute physical performance indicators and the number of squad inclusions in the first five official matches. For this analysis, squad inclusions were counted for each player as the total number of matches in which he appeared on the official squad list, ranging from zero to five. Considering this restricted range, the associations were interpreted cautiously. Higher pre- and post-assessment values for SJ, CMJ, COD-20 m, and the composite physical score were associated with a greater number of squad inclusions. The highest coefficient was observed between post-assessment COD-20 m and the number of squad inclusions.

Table 4 presents the complementary comparison of pre-to-post change scores between squad-included and non-included players. This analysis was performed to verify whether squad-included players showed greater pre-to-post changes during the pre-season period. No pre-to-post change score differed significantly between groups. The lowest *p*-value was observed for the COD-20 m change score, although it did not reach statistical significance.

Pre-assessment maturity offset from peak height velocity was analyzed as a complementary variable to verify whether competitive involvement was accompanied by differences in estimated biological maturation. Maturity offset did not differ between squad-included and non-included players (0.40 ± 0.55 vs. 0.28 ± 0.87 years; *p* = 0.617; d = 0.16) and was not significantly associated with the number of squad inclusions in the first five official matches (ρ = −0.03; *p* = 0.844). These results indicate that, in this sample, initial competitive involvement was not accompanied by statistically identifiable differences in estimated biological maturation.

## 4. Discussion

The present study examined pre-season physical-performance changes and their relationship with early competitive squad inclusion in under-15 male soccer players. The main finding was that squad-included players presented higher absolute physical performance at both assessment moments, with the clearest individual-test difference observed for post-assessment COD-20 m. In contrast, the magnitude of pre-to-post change did not differ significantly between squad-included and non-included players, and estimated maturity offset was not associated with early squad inclusion. These findings partially support the study hypothesis and indicate that, in this applied academy context, current physical-performance status was associated with early squad inclusion, whereas short-term physical change did not differ significantly between groups.

The overall physical-performance changes observed across the pre-season period were selective, with significant increases in SJ, CMJ, and COD-20 m, whereas Sprint-20 m and Yo-Yo IRL1 remained unchanged. This pattern is consistent with previous evidence suggesting that pre-season training preferentially enhances neuromuscular qualities, particularly those related to lower-limb strength and power, while adaptations in sprint and aerobic performance may require different training stimuli or longer exposure [26]. The increase in COD-20 m performance is plausible because short-term change-in-direction interventions or exposure to many changes of direction as in small games can improve cutting completion time, change-in-direction deficit, and movement quality in male youth soccer players [27,28]. The lack of significant linear-sprint change may reflect the task specificity of adaptation, because studies in youth soccer indicate that linear and multidirectional speed tests are related but not interchangeable performance constructs [29]. The absence of significant Yo-Yo IRL1 improvement should also be interpreted cautiously, because Yo-Yo IR1 performance is reliable in youth soccer but still shows test–retest variability that can obscure small short-term changes in applied samples [30]. The statistically significant group-level changes in SJ, CMJ, and COD-20 m, considered together with their corresponding effect-size estimates, support changes at the sample level. However, because test-specific typical error was not estimated in the present sample, whether individual changes exceeded expected measurement variability could not be determined. These results indicate higher post-assessment performance in the jumping and change-in-direction tests, whereas changes in aerobic-intermittent and linear-sprint performance were not statistically significant.

The between-group findings indicate that squad-included players were already physically advantaged before the start of official competition and maintained or amplified this advantage at post-assessment. These findings are consistent with a systematic review indicating that physiological and physical characteristics have prognostic value in youth soccer, while emphasizing that the strength and consistency of this evidence vary across studies and performance outcomes [3]. They also support the notion that progression to elite youth soccer is a multidimensional process that cannot be explained by isolated physical attributes alone [1]. Coach-based talent-identification research similarly indicates that practitioners consider technical, tactical, psychological, perceptual, social, and physical criteria when evaluating 13- to 16-year-old male players [31]. The association between stronger physical profiles and early squad inclusion is therefore best interpreted as evidence that physical readiness contributed useful information in this setting, not as evidence that physical testing alone determined squad selection. This interpretation is reinforced by recent youth soccer research showing that match participation is related to combinations of maturation, fitness, and hormonal variables rather than to any single isolated determinant [17]. Therefore, the present study extends prior work by linking pre-season field-test data to early official squad-list inclusion rather than to broader season-long or preseason playing-time indicators.

The largest individual-test difference between squad-included and non-included players was observed for post-assessment COD-20 m, and post-assessment COD-20 m showed the strongest association with the number of squad inclusions. This finding is coherent with evidence that change-in-direction ability in young soccer players is associated with sprinting, strength, and power capacities, but is not fully explained by those qualities alone [32]. Age-specific youth soccer data also indicate that change-in-direction performance and change-in-direction deficit follow developmental patterns that are distinct from straight sprinting and jumping performance [33]. Change-in-direction tasks require deceleration, braking control, body reorientation, lateral force application, and reacceleration, which helps explain why they may capture a more integrated physical quality than straight-line speed alone [27]. The stronger relevance of COD-20 m at post-assessment may indicate that the final pre-competition testing moment better represented the physical state available to coaches when early squad decisions were made.

The absence of significant between-group differences in pre-to-post changes suggests that squad-included players were not simply those who improved the most during the monitored pre-season period. However, given the relatively small and unequal groups, these findings should be interpreted as an absence of statistically detectable differences in the present sample rather than as definitive evidence of equivalence between the groups. The effect-size estimates provide complementary information about the magnitude of the observed differences. This finding is important because field-test change scores in youth soccer should be interpreted relative to measurement reliability, biological variability, and baseline performance level [4]. Yo-Yo test performance generally shows good-to-excellent test–retest reliability, but evidence still supports the need to consider typical error when interpreting individual or small-group change [34]. Because training content, volume, intensity, session attendance, and internal and external training loads were not quantified, it was not possible to determine whether players received comparable training stimuli or to attribute the observed changes specifically to the pre-season training process. Accordingly, the current results support the practical distinction between current physical status and short-term physical change, because early squad inclusion appeared to track the former more clearly than the latter.

The lack of association between estimated maturity offset and early competitive involvement contrasts with much of the adolescent athlete literature, but it should not be interpreted as evidence that maturation is irrelevant in youth soccer. A systematic review and meta-analysis reported meaningful relationships between biological maturation, physical fitness, and kinanthropometric variables in young athletes [13]. In academy soccer, maturity status has been shown to have a stronger association than relative age with sprint performance in male players [14]. In under-15 soccer players, match participation has also been associated with the combined influence of maturity status, physical fitness, and hormonal indicators [17]. The present null finding may therefore reflect the specific outcome used in this study, the restricted early-competition follow-up, the relatively small and homogeneous sample, or the narrow distribution of maturity offset around peak height velocity. It may also reflect limitations of anthropometric maturity prediction, because the original maturity-offset equation provides a practical non-invasive estimate but was not designed to be an error-free individual classification tool [19]. Recent soccer validation work has questioned the utility of predicted maturity offset and predicted age at peak height velocity for precise maturity timing and status classification in individual players [20]. More recent evidence in elite youth soccer also shows imperfect concordance between predicted maturity-status classifications and observed peak-height-velocity timing [35]. These considerations indicate that the maturity result should be interpreted as a specific absence of a detectable association with early squad inclusion, not as a general rejection of maturational influences in youth soccer.

Limitations should be considered when interpreting these findings. The sample included male outfield players from one under-15 program, and the squad-included and non-included groups were relatively small and unequal, reducing the precision of between-group estimates. Positional composition also differed between groups and may have partly influenced the observed physical-performance differences; however, the small number of players within each position did not support position-stratified analyses. Given the number of comparisons and correlations, individual *p*-values should be interpreted alongside effect sizes and the overall pattern of findings. Squad inclusion was based on official match reports and did not indicate whether players entered the match, accumulated playing time, or contributed to match performance; therefore, it should be interpreted as squad-list inclusion rather than competitive participation. The observational design and absence of a control group prevent causal attribution of the observed changes to the monitored pre-season period. Training content, volume, intensity, session attendance, and internal and external loads were not quantified, limiting interpretation of adaptation, non-response, and dose-response relationships. Maturity was estimated from anthropometry rather than longitudinal or skeletal-age methods. The composite physical score was an exploratory, non-validated summary measure; its’ equally weighted components may represent related physical-performance characteristics, and its measurement properties were not established in this sample. Future studies should use larger multicenter samples, repeated assessments across the competitive season, direct maturity indicators, load monitoring, injury and availability records, minutes played, and technical–tactical data. Multivariable analyses should also examine interactions among physical performance, maturation, playing position, coach evaluation, and match context.

From an applied perspective, the findings support the use of pre-season physical testing as part of a broader readiness-monitoring process in youth soccer. The strongest practical message is that absolute physical performance, especially lower-limb power, change-in-direction performance, and an integrated physical profile, may help practitioners identify physical-performance characteristics associated with early squad inclusion. This interpretation is consistent with evidence supporting the reliability and validity of commonly used field-based fitness tests in youth soccer, provided that tests are selected and interpreted carefully [4]. However, physical testing should complement rather than replace coach judgment because talent identification and squad decisions in youth soccer are multidimensional [1]. The absence of stronger associations for change scores suggests that practitioners should avoid interpreting short-term improvement alone as evidence of competitive readiness. Instead, pre-season monitoring may be most useful when current physical status, maturation, training exposure, wellness, technical–tactical performance, and contextual information are considered together.

## 5. Conclusions

Significant increases in SJ, CMJ, and COD-20 m were observed across the monitored pre-season period, whereas Sprint-20 m and Yo-Yo IRL1 did not change significantly. Squad-included players presented higher absolute physical performance at both assessment moments, with the most pronounced individual-test difference and strongest association with the number of squad inclusions observed for post-assessment COD-20 m. In contrast, pre-to-post change scores and estimated maturity offset were not clearly associated with early squad inclusion. These findings indicate that, in this applied academy sample, early squad inclusion was associated with higher current physical-performance levels, particularly post-assessment COD-20 m, while reinforcing that physical monitoring should be interpreted as one component of a multifactorial youth soccer decision-making process.

## Figures and Tables

**Table 1:** Changes in physical performance between pre- and post-assessments in the overall sample.

Variable	Pre	Post	Mean Δ	*p*	Effect Size
SJ (cm)	32.06 ± 5.19	33.50 ± 4.78	+1.44	0.007 *	0.51
CMJ (cm)	33.29 ± 5.53	34.65 ± 5.31	+1.36	0.006 *	0.49
Sprint-20 m (km·h^−1^)	23.08 ± 1.32	22.98 ± 1.37	−0.11	0.363	−0.15
COD-20 m (km·h^−1^)	13.32 ± 0.62	13.70 ± 0.76	+0.38	0.002 *	0.60
Yo-Yo IRL1 (m)	1711.89 ± 485.83	1635.14 ± 564.57	−76.76	0.384	−0.15

Note: Values are presented as mean ± standard deviation. Δ = post − pre. SJ = Squat Jump; CMJ = Countermovement Jump; Sprint-20 m = 20 m linear sprint; COD-20 m = 20 m change-in-direction test; Yo-Yo IRL1 = Yo-Yo Intermittent Recovery Test Level 1. Sprint-20 m and COD-20 m were expressed as mean velocity; therefore, higher values indicate better performance in all physical variables. Cohen’s d was used for paired *t*-tests, and rank-biserial correlation was used for Wilcoxon signed-rank tests. Effect sizes are presented according to the direction of Δ. * *p* < 0.05.

**Table 2:** Comparison of pre- and post-assessment physical performance between squad-included and non-included players.

Variable	Time Point	Squad-Included (*n* = 14)	Non-Included (*n* = 23)	*p*	Effect Size
SJ (cm)	Pre	34.85 ± 4.17	30.36 ± 5.08	0.006 *	0.97
	Post	35.97 ± 4.78	32.00 ± 4.21	0.017 *	0.88
CMJ (cm)	Pre	35.95 ± 4.89	31.67 ± 5.35	0.019 *	0.84
	Post	37.58 ± 5.92	32.87 ± 4.09	0.016 *	0.93
Sprint-20 m (km·h^−1^)	Pre	23.54 ± 0.92	22.80 ± 1.46	0.071	0.60
	Post	23.54 ± 1.00	22.63 ± 1.47	0.031 *	0.73
COD-20 m (km·h^−1^)	Pre	13.64 ± 0.56	13.12 ± 0.59	0.013 *	0.89
	Post	14.19 ± 0.49	13.40 ± 0.74	<0.001 *	1.25
Yo-Yo IRL1 (m)	Pre	1731.43 ± 485.59	1700.00 ± 496.50	0.851	0.06
	Post	1770.00 ± 543.99	1553.04 ± 572.80	0.258	0.39
Composite physical score	Pre	0.38 ± 0.63	−0.23 ± 0.71	0.010 *	0.92
	Post	0.68 ± 0.51	−0.12 ± 0.67	<0.001 *	1.35

Note: Values are presented as mean ± standard deviation. SJ = Squat Jump; CMJ = Countermovement Jump; Sprint-20 m = 20 m linear sprint; COD-20 m = 20 m change-in-direction test; Yo-Yo IRL1 = Yo-Yo Intermittent Recovery Test Level 1. Sprint-20 m and COD-20 m were expressed as mean velocity. The composite physical score was calculated as the mean of the z-scores for SJ, CMJ, Sprint-20 m, COD-20 m, and Yo-Yo IRL1, using the pre-assessment mean and standard deviation as reference values. This score should be interpreted as an exploratory standardized summary of the overall physical profile, not as a previously validated performance index. Cohen’s d was used as the effect size for Welch’s *t*-tests. Positive effect sizes favor squad-included players. * *p* < 0.05.

**Table 3:** Correlations between absolute physical performance and number of squad inclusions in the first five official matches.

Variable	Time Point	ρ with Squad Inclusions	*p*
SJ	Pre	0.42	0.010 *
	Post	0.36	0.027 *
CMJ	Pre	0.34	0.040 *
	Post	0.42	0.009 *
Sprint-20 m	Pre	0.26	0.119
	Post	0.27	0.103
COD-20 m	Pre	0.43	0.007 *
	Post	0.58	<0.001 *
Yo-Yo IRL1	Pre	−0.05	0.780
	Post	0.14	0.399
Composite physical score	Pre	0.38	0.019 *
	Post	0.53	<0.001 *

Note: ρ = Spearman’s rank correlation coefficient. Squad inclusions = number of matches in which the player was included in the official squad list across the first five official matches. Positive coefficients indicate that higher physical performance values were associated with a greater number of squad inclusions. * *p* < 0.05.

**Table 4:** Complementary analysis of pre-to-post changes between squad-included and non-included players.

Variable	Δ Squad-Included (*n* = 14)	Δ Non-Included (*n* = 23)	*p*	Effect Size
SJ (cm)	1.12 ± 3.29	1.64 ± 3.29	1.000	0.00
CMJ (cm)	1.63 ± 2.57	1.20 ± 2.99	0.651	0.15
Sprint-20 m (km·h^−1^)	0.01 ± 0.68	−0.17 ± 0.71	0.331	0.20
COD-20 m (km·h^−1^)	0.55 ± 0.50	0.28 ± 0.68	0.062	0.37
Yo-Yo IRL1 (m)	38.57 ± 616.02	−146.96 ± 470.34	0.424	0.16
Composite physical score	0.30 ± 0.36	0.11 ± 0.50	0.193	0.43

Note: Values are presented as mean ± standard deviation. Δ = post − pre. SJ = Squat Jump; CMJ = Countermovement Jump; Sprint-20 m = 20 m linear sprint; COD-20 m = 20 m change-in-direction test; Yo-Yo IRL1 = Yo-Yo Intermittent Recovery Test Level 1. Sprint-20 m and COD-20 m were expressed as mean velocity; therefore, positive Δ values indicate higher post-assessment performance. Cohen’s d was used as the effect size for Welch’s *t*-tests, and rank-biserial correlation was used for Mann–Whitney U tests. Positive effect sizes favor squad-included players.

## Data Availability

The data presented in this study are available upon reasonable request from the corresponding author. The data are not publicly available due to privacy and ethical restrictions related to the potential identification of the under-15 athletes included in the study.
